# “There’s a Lot of Like, Contradicting Stuff”—Views on Healthy Living during Pregnancy and Postpartum

**DOI:** 10.3390/ijerph19105849

**Published:** 2022-05-11

**Authors:** Jade A. McNamara, Noereem Z. Mena, Arrington Wright, Makenzie L. Barr

**Affiliations:** 1 Department of Food Science and Nutrition, University of Maine, Orono, ME 04473, USA; jade.mcnamara@maine.edu; 2 Division of Nutritional Sciences, Cornell University, Ithaca, NY 14850, USA; noereem.mena@cornell.edu; 3 Department of Dietetics and Human Nutrition, University of Kentucky, Lexington, KY 40506, USA; arrington.wright@uky.edu

**Keywords:** pregnancy, postpartum, nutrition, mental health, support, qualitative

## Abstract

The transition from pregnancy through early postpartum can be a particularly vulnerable time for women as they adjust to the changes of motherhood. This study aimed to provide a detailed account of additional health challenges that mothers are facing throughout motherhood during the pandemic. Data obtained can be utilized to create tailored interventions to aid women during their reproductive years. A sequential approach was utilized, collecting health-related information via survey and subsequent focus groups or interviews to further examine health experiences during pregnancy or postpartum. Fifty-seven participants completed the online survey, 73.5% were postpartum. The healthy eating index of the cohort was low, 50.5 ± 10.3%. Prior to pregnancy, 54.5% were classified as overweight/obese. Following pregnancy, 71.1% were classified as overweight or obese. Emergent qualitative themes from focus groups (n = 3) and interviews (n = 6) included (1) value and desire for healthy eating, (2) desire to make well-informed health-based decisions, and (3) role of social networks during pregnancy and postpartum. Pregnant/postpartum women desire to lead a healthy lifestyle but experience barriers to accomplishing intended goals. Upstream resources and policies that promote healthy living for pregnant/postpartum women can reduce chronic disease throughout the lifespan following childbirth.

## 1. Introduction

The transition from pregnancy through early postpartum can be a particularly vulnerable time for women as they adjust to the changes of motherhood. Mothers often experience immense biological, emotional, financial, and social changes during this time, yet many report feeling unprepared for postpartum care and lack a recovery plan [1]. However, motherhood can be an impressionable time, where dietary behaviors and nutritional knowledge can be formed or adjusted to reduce the risk of obesity or related chronic disease postpartum [2]. During this life event, a window of opportunity opens whereby a mother is increasingly motivated to learn healthy eating practices to impede inappropriate weight gain, lessen postpartum weight retention, and promote the health of their baby [2]. Though many pregnant women in the U.S. fail to meet several established recommendations for micronutrients as well as exceeding suggested intakes for sodium [3]. A study in 2017, examining the preconceptual healthy eating index of pregnant women, found subpar nutritional intake among participants [4,5]. Scores were found to fall below recommendations set forth in the Dietary Guidelines for Americans [6] and over one-third of all reported intakes were noted to be from empty non-nutritive calories [4].

Quality nutrition and physical activity are paramount to ensure the well-being of both mother and child, as the fetal environment plays a pivotal role in the lifelong health status of a newborn [7,8,9]. In the United States, almost one-third of pregnancies begin with a mother who is classified as overweight or obese by body mass index (BMI) [10,11,12,13,14]. Long-term health outcomes for both mother and child can be influenced largely beginning in periconception, through delivery and beyond [15]. Approximately half of women will gain weight that exceeds recommendations and will retain that weight at one-year postpartum [16]. For women who enter pregnancy with a healthy BMI, approximately one-third will become overweight or develop obesity by 12 months postpartum [17,18]. Managing weight before, during, and after pregnancy is crucial to inhibit the development or continuation of obesity [19,20], and decrease the risk of developing additional related health conditions, later in life [21].

While postpartum check-ups and health care appointments traditionally assess infant growth and health, mothers receive limited and infrequent care for their own well-being [22,23]. Women across all maternal groups describe how the postpartum period brought about significant changes and challenges, including exhaustion, stress, poor body image, and marital discord [24]. Likewise, time constraints and prioritization of maternal responsibilities tend to come before their own personal health [25]. Multiple studies have reported that pregnant and postpartum women receive inadequate information regarding nutrition, well-being, and weight status throughout pregnancy and the postpartum period [26,27], indicating a need for clearer guidelines and recommendations. For many individuals seeking health-related information throughout the journey to, and through motherhood, inundation of online feedback is mainstream. The use of technology-based approaches for seeking information is commonplace; however, it could be detrimental, and often contradictory.

Research exists on understanding the health and well-being of women during pregnancy and the postpartum period [25]; however, to ensure the personal health of mothers throughout this journey, research is needed to assess individual-level maternal health needs throughout this life stage. The current study aimed to utilize a sequential approach to collect basic quantitative data and subsequent qualitative data to gain insight into the cognitive, behavioral, and environmental influences of healthy dietary practices among pregnant and postpartum women. Data collection was conducted during the coronavirus 2019 (COVID-19) pandemic, which has been shown to heighten poor outcomes [28] and reduce access to helpful resources for women throughout motherhood [29]. The current study aims to provide a detailed account of additional health challenges that mothers are facing throughout motherhood during the pandemic. The authors hypothesize that pregnant and postpartum individuals will highlight the need for more thorough health-related support than they currently receive throughout this journey. Data obtained can be utilized to create tailored interventions to aid women during their reproductive years.

## 2. Materials and Methods

Biological females, 18 years and older, English speaking, who were currently pregnant or within one-year postpartum, were recruited to participate in this study to complete a cross-sectional online survey and a follow-up interview or focus group session. All study procedures were approved by the University of Kentucky and University of Maine Institutional Review Boards.

Participants were recruited via social media advertisements including an anonymous, online, Qualtrics [30] survey link (Supplementary Figure S1). Inclusion criteria included individuals who were 18 years of age or older, currently pregnant or within one-year postpartum, able to read, understand, and speak the English language, and able to utilize online technology to complete study data collection. Volunteers self-selected to participate in the online survey that collected demographic information (age, race, ethnicity, residency location, relationship status, and education level), and number of pregnancies (e.g., gravidity). Health-related information was collected on body mass index (BMI) before their first pregnancy, current BMI if postpartum, and the short healthy eating index (sHEI) tool was used to assess dietary quality. The sHEI is a 22-item tool to assess overall dietary quality and estimate consumption of some individual food components [31]. The tool provides a score that is a percentage between 0 and 100, with higher numbers indicating a healthier dietary quality intake, i.e., higher scores indicate a dietary pattern more in line with the Dietary Guidelines for Americans.

Following the online survey, participants were given the opportunity to self-select to participate in a focus group session lasting approximately 60 min. The Zoom online video platform was used to conduct and record focus group sessions. Individuals willing to participate were sent instructions on navigating the platform and the time and date of their scheduled focus group. Verbal consent was obtained prior to each focus group session by the researcher. Sessions were lead by a single experienced, trained, interviewer. Those willing to complete the focus group were compensated with a gift card for their time.

### 2.1. Development of Focus Group and Interview Guide

Informed by Social Cognitive Theory (SCT) [32], a semi-structured interview guide was developed to capture cognitive/personal factors (knowledge, expectations, and attitudes), behavioral factors (skills and self-efficacy), and environmental factors (social norms, access, and influences) related to eating and other health-related behaviors of pregnant and postpartum women. Questions were drafted by one qualitative researcher and reviewed by two additional qualitative researchers. The final interview guide contained 11 questions for interviews to be conducted with pregnant women, and 10 questions for interviews to be conducted with postpartum women (Table 1).

A total of 3 interviews and 1 focus group (n = 3) were conducted with pregnant women, and a total of 3 interviews and 2 focus groups (n = 2) were conducted with postpartum women.

### 2.2. Data Analysis

Audio recordings were transcribed verbatim by the Zoom online platform and subsequently verified by a member of the research team. Transcripts were then de-identified and made available to a third researcher, trained in qualitative data collection methodologies and analyses for coding and thematic analysis. Using the moderator guide as a guide, a codebook was developed with a priori codes to qualitatively capture cognitive, behavioral, and environmental factors that impact eating and other health-related behaviors among pregnant and postpartum women.

One transcript from each group was randomly selected to confirm a priori codes via supportive text segments. Emergent codes were identified and added to the codebook. Remaining transcripts were coded using the final codebook and modified as needed throughout the coding process. A directed and deductive content analysis approach was utilized as our primary analytic technique [33], using SCT constructs as a guide to identify factors that influence pregnancy and postpartum healthy dietary practices.

## 3. Results

Of participants who completed the online survey (n = 57), 73.5% were postpartum (n = 39), between 20 and 34 years of age (28.2 ± 3.6SD), 93.0% were married, and 98.2% were white. The highest frequency of household income grouping was between 100,000 and 150,000 USD (n = 17; 29.8%), 54.7% identified as residing in a rural area, and there was equal representation across four levels of education status (Table 2). Participants had an average of two pregnancies (2.06 ± 1.09SD). Among demographic variables, no significant differences were seen between currently pregnant participants and those within one-year postpartum (all *p* > 0.05).

Among health status measures, the sHEI score was an average of 50.5 ± 10.3% for the full cohort (54.0 ± 10.3% among currently pregnant women and 48.9 ± 9.9% among postpartum women; *p* = 0.06). Of women with complete height and weight data, prior to pregnancy, 54.5% were classified as overweight or obese by BMI ≥ 24.9 kg/m2. Following pregnancy, for those up to one-year postpartum, 71.1% are currently classified as overweight or obese.

### 3.1. Qualitative Results

Overall, participants in both the pregnant and postpartum groups reported feeling mostly confident in being able to make healthful food choices, although this did not always necessarily translate into actual behavioral decisions. Additionally, information related to overall health promotion during pregnancy and postpartum felt limited—participants reported yearning for more information. Structural and environmental barriers to healthy eating (i.e., access to a variety of healthful foods) were uncommon, although interruptions to the food system due to the COVID-19 pandemic was reported by one participant as impacting the availability and variety of fresh produce at the supermarket. Participants actively sought information related to promoting health and nutrition during pregnancy and postpartum, and a couple of participants mentioned using health-related pregnancy and postpartum mobile applications to help with informed decision making.

Three major themes emerged across the SCT constructs with subsequent supporting themes (Table 3). Value and desire for healthy eating during pregnancy and the postpartum period emerged as a major theme related to cognitive/personal factors that influence healthy dietary practices during pregnancy and the postpartum period. Two related subthemes also emerged: influence on baby’s health and diet quality is compromised in early postpartum. Desire to make well-informed health-based decisions emerged as a major theme related to behavioral factors that influence healthy dietary practices during pregnancy and the postpartum period. Ability to navigate the internet and social media to access desired information, and holistic approach to pregnancy and the postpartum period emerged as two supporting subthemes for behavioral factors. Related to environmental factors, the role of social networks during pregnancy and the postpartum period emerged as a major theme and COVID-19 pandemic impact on social networks emerged as a subtheme.

### 3.2. Cognitive/Personal Factors

#### 3.2.1. Theme 1: Value and Desire for Healthy Eating during Pregnancy and the Postpartum Period

Healthy eating habits were viewed as important for health promotion during pregnancy and postpartum. Some participants described their pre-pregnancy eating habits as relatively healthy; however, once in pregnancy, there was a conscious effort to make more healthful food choices. Diet variety and adequate intake of fruits and vegetables were reported as characteristics of a healthy diet during pregnancy, and for some, changes in dietary habits during pregnancy carried over into the postpartum period. For one pregnant participant specifically, there was an increased emphasis on buying foods of higher quality, which was further reinforced by how changes also made her feel physically (Table 3).

Pregnant and postpartum individuals reported various reasons to engage in healthy eating habits. Reasons included food safety, i.e., avoiding foods deemed ‘not safe’ for consumption during pregnancy, medical conditions such as gestational diabetes and constipation, and making pregnancy more comfortable. Pregnancy seemed to motivate individuals to make better food choices, or what some described as doing what was in the “best interest of the baby”, although one (pregnant) participant, in a moment of reflection, stated, “prepare yourself a little more, start eating healthier before [getting pregnant], instead of waiting till your mid pregnancy trying to change your habits”. Overall, few pregnant individuals reported barriers to healthy eating, but they did acknowledge instances of prioritizing convenience over quality nutrition due to a lack of time, energy, or limited support. One participant stated, “it’s just [me] not wanting to make healthy food because healthy food is often not as convenient as unhealthy food” (pregnant woman). Sacrificing nutrition and quality for convenience became more pronounced during the early postpartum period (see Subtheme 1.2).

##### Subtheme 1.1: Influence on Baby’s Health

Participants were also aware of the influence their dietary habits and intakes had on the health of their unborn offspring, and dietary habits of their infants and toddlers. Pregnant individuals described making healthful changes “for the baby”, and avoiding foods that could potentially lead to food-borne illness (Table 3). Among postpartum individuals, if breast feeding, changes in dietary habits were due to increased energy needs, ensuring adequate supply of breastmilk, and baby food sensitivities. Moreover, the importance of offering more healthful foods and role modeling healthful dietary habits as infants transitioned to table foods emerged as a driver in food-based decisions. For some, their infant’s transition to complementary feeding and table foods helped facilitate or provide additional motivation for healthy eating as mothers typically served their infant/toddlers the same foods they served themselves and vice versa. One postpartum participant talked about despite being a “terrible cook”, and because that influenced what she and her family ate, she was motivated to learn how to incorporate healthy food options for her family.

##### Subtheme 1.2: Diet Quality Is Compromised in Early Postpartum

Sustaining healthy eating routines early in the postpartum period was challenging for postpartum individuals. Specifically, the first three months were described as hectic, overwhelming, and “just pretty much surviving” as one (postpartum) participant put it. A few mentioned an awareness of or need for increased nutrient needs; however, disruptions in “normal” routines and a lack of time and energy made it difficult to establish routines that allowed consistent eating routines or ensure adequate intake. Postpartum individuals reflected on a greater emphasis on eating as healthily as possible during pregnancy; however, once in the postpartum period, convenience over quality was prioritized. Furthermore, the postpartum period lead some to feel less anxious and “freer” regarding food choices and dietary intake as they no longer had to worry about avoiding foods deemed unsafe for consumption during pregnancy or eating something that could potentially harm their unborn baby (Table 3).

### 3.3. Behavioral Factors

#### 3.3.1. Theme 2: Desire to Make Well-Informed Health-Based Decisions

Participants expressed frustrations over the limited guidance provided and available to them during pregnancy and the postpartum period. Although physicians eased concerns related to the pregnancy and postpartum experience, there was a consensus across both the pregnant and postpartum groups that information provided from their physician, although helpful, was often limited (Table 3). Information provided related to nutrition primarily focused on food safety (i.e., avoiding foods not safe for consumption during pregnancy, rarely on promoting diet quality). During the postpartum period, there was a desire for more information on expectations (what is ‘normal’) and ensuring adequate nutrient intake. While a couple of the participants were familiar with the term ‘registered dietitian’, none had any formal interactions with a registered dietitian, except for one participant who was referred to a registered dietitian by her physician due to child feeding and nutrition concerns.

##### Subtheme 2.1: Need for Comprehensive Holistic Approach to Pregnancy and Postpartum Health

While participants felt that their concerns related to pregnancy and the postpartum period were eased when able to communicate with their physician, it lacked a holistic approach to health promotion during these critical periods. Information provided rarely discussed other aspects of health, including physical, emotional, and mental health. Few discussed the role of physical activity in their pregnancy and/or the postpartum period, but one participant did state an awareness of the benefits of physical activity for unborn offspring, and therefore it became “really important” to continue to engage in physical activity during pregnancy. This participant also reported paying for a mobile app subscription to receive information related to not only nutrition but “pregnancy friendly activities”.

Participants in both the pregnancy and postpartum groups expressed a need for a greater emphasis on mental health and overall expectations for pregnancy and the postpartum period. This type of information was referred to by one pregnant participant as “helpful” and by a postpartum participant as reassurance that what she was experiencing was normal. As she put it “I wish there was just a pamphlet of stuff [saying] this is very normal [during] postpartum”. Pregnant participants described feelings of anxiety, rooted in eating something that would harm their unborn offspring. Postpartum participants talked about feeling overwhelmed with the challenges of adjusting to having a newborn and work–life balance. Interrupted sleep cycles specifically during the postpartum period compounded feelings of stress for some, even impacting their mood. As one postpartum participant stated, “My moods definitely are a struggle. During the day I am fine but obviously at night, I am the only person that can feed her [daughter], I’m the only one that’s getting up in the middle of the night. It’s going to be much more of a struggle when I go back to work, which I am dreading”. The COVID-19 pandemic was also reported to impact mental health (Table 3).

Postpartum participants expressed wanting more guidance and support in navigating the postpartum journey (Table 3). A couple of postpartum participants felt that there was a greater emphasis on the health and nutritional status of the infant given the number of follow-up doctor visits an infant has in the first few months of life, in comparison to the one (and often not enough) 6 week postpartum follow-up visit for mothers. Several felt that the 6 week follow-up visit was simply not enough, particularly to help them understand what was happening to their body and how to ensure adequate nutrient intake during postpartum. One participant in particular expressed concern over the possibility of having micronutrient deficiencies, but simply being unsure.

##### Subtheme 2.2: Ability to Navigate the Internet and Social Media to Access Desired Information

Participants in both the pregnant and postpartum groups expressed a desire to easily access credible, evidence-based information, and many relied on the internet or social media for information. While seeking information online was a frequent practice, it, at times, became burdensome due to outdated or inaccurate information related to pregnancy or postpartum (Table 3). Google was reported by a couple of postpartum participants as their primary source engine. How the information was presented, or content tone, was also important. An emphasis on what was normal or expected during pregnancy and postpartum, and realistic, applicable recommendations were also identified as how participants wanted information presented. What was currently available seemed unrealistic to attain, as one pregnant participant stated, “If I was going to follow the instructions on like ‘what to expect when expecting,’ I was like man this feels extreme compared to how I eat”. The use of social media (i.e., following accounts on Instagram and Facebook) for postpartum participants primarily focused on information related to feeding infants and toddlers.

### 3.4. Environmental Factors

#### 3.4.1. Theme 3: The Role of Social Networks during Pregnancy and Postpartum

Social networks were important for both pregnant and postpartum participants. Social networks were a source of support and provided information to support healthy eating habits (Table 3). Participants sought advice from their doctors, family and peers, and a supportive spouse within the home was also reported to be extremely helpful. Spouse and peers were reported to influence eating and activity behaviors for pregnant and postpartum participants. For postpartum participants, being open to asking for and accepting help was crucial. A crucial timepoint for support was described as “the first six months”. Assistance with meal preparation and cooking was described by one participant as extremely valuable. The COVID-19 pandemic caused disruptions and interruptions to social and cultural expectations for pregnant and postpartum participants.

##### Subtheme 3.1: COVID-19 Pandemic Impact on Social Networks

The COVID-19 pandemic impacted social expectations and traditions of pregnancy and postpartum. The lockdown and social distancing measures impacted the ability to have in-person events. Health concerns were reported as well. As one pregnant participant stated, “I worry about it (COVID-19) the entire time because we were in the swing of COVID when I found out I was pregnant. I pretty much never leave the house”. Other COVID-19 impacts included not being able to bring their spouse to doctor’s appointments or connect with peers and limited parent–infant social interactions. These impacts lead some participants to feel socially isolated and left with unmet expectations (Table 3).

## 4. Discussion

The purpose of this paper was to explore the cognitive, behavioral, and environmental influences of healthy dietary practices among pregnant and postpartum women. This study found that the three constructs within the SCT all played a role in how women navigate their health and health decision making while pregnant/postpartum. Self-reported behaviors showed an overall poor diet quality score, especially when comparing pregnant women to postpartum women. Low diet quality scores were further explained during the focus groups as women expressed challenges with putting their desires for healthy eating into practice. While the focus group questions were directed towards uncovering themes related to eating behavior, there was an underlying theme of mental health that arose throughout all the conversations. The findings from the current study build upon the importance of understanding the factors and experiences that new mothers are facing when managing their own health during pregnancy and postpartum.

As evident by the themes that emerged, women placed strong value in healthy eating during pregnancy and were invested in learning about and meeting the dietary guidelines for Americans [6], emphasizing foods that promote diet quality and are beneficial for their developing baby, and they were willing to try new foods and activities that they knew to be healthy. The pregnancy group did report a sHEI score that was below ideal, likely as a result of the many challenges women who are pregnant face even when they are feeling highly motivated to eat healthily.

A compromised diet may be especially evident during the postpartum period. Women in this study expressed that during the early postpartum period, they were “just surviving”, and that since having a baby, “it’s like who knows when any meal is going to be”. This theme of diet quality compromised in early postpartum aligns with the dietary data in that a lower, but not significant, sHEI score was found in the postpartum group verses the pregnancy group. This is consistent with a previous study conducted by Martin et al. 2020 [34], who found a significant decrease in diet quality score as more time went on since childbirth. In their longitudinal study (N = 4539), women reported the lowest diet quality at >12 months postpartum when compared to 0–6 months and 7–12 months. This is concerning given that diet quality is already low during pregnancy, and then decreases further as their children age, putting mothers at greater risk of unwanted weight gain, poor mental health, and development of chronic disease [19,20,21]. Further research examining how to improve maternal diet quality is warranted to ensure best health outcomes during the first 1000 days.

Another major theme that emerged was women reporting feeling overwhelmed when trying to make well-informed healthy eating decisions. Many quotes support the notion that making informed decisions was difficult during pregnancy because doctors did not share many resources that focused on nutrition and mental health. Even with most of the sample having an associate degree or higher and making 75,000 USD a year or more, aspects that are associated with better health outcomes [35], participants still reported struggling with feeling confident in their behavior choices. These findings reflect those that Pullon et al. 2018 found, where pregnant women expressed frustration with information overload when making decisions about nutrition and food choices [36]. This underscores the importance of providing clear and concise recommendations to pregnant and postpartum women on a regular basis to help them navigate their food environments.

The participants recount that social support was crucial to their well-being, especially during the COVID-19 pandemic, citing experiences such as having a friend who was also pregnant, being part of a virtual group of women who had babies that were the same age, and having a spouse that helped to make healthy choices easier (cooking, walking, etc.). The importance of women having social support during pregnancy, especially while living through a pandemic, is confirmed by Khoury et al., who found that mental health-related issues in pregnant women are more prevalent since the COVID-19 pandemic when compared to pre-pandemic data due to the risk of infection, social isolation, relationship difficulties, and financial hardships [37]. Contrary to these findings, Silverman et al. showed that based on medical records collected during the stay-at-home order for COVID-19, low-income postpartum women reported less mood disorder symptoms than high-income women [38]. The authors explained this outcome by stating that low-income women may not be experiencing the normal stressors that they may usually face such as unavailable childcare, limited support from their partner, and multiple job obligations since people were being forced to stay at home. Collectively, these findings underscore the importance of focusing on bettering the mental health of mothers during pregnancy and postpartum.

Findings from this study are supported by similar outcomes to Makama et al. [39] and Ryan et al. in 2021 [25]. The authors conducted systematic reviews of qualitative and quantitative studies that indicated areas of postpartum care that were facilitators or barriers to healthy living. Social support, a lack of time and motivation, inadequate knowledge transfer from health care professionals, and prioritization of childcare needs were all identified as areas of influence for achieving a healthy lifestyle throughout the pregnancy and postpartum journey. Although individuals must take personal responsibility for behavior changes and sustainability, the community and systems in place to support these changes are inevitably influential. Our findings add to the literature regarding the needs of mothers in terms of supporting their health as well as the adjustments made throughout COVID-19.

### Limitations

This study is not without limitations. Participants were a convenience sample of those interested in participating in a study such as this. Women self-selected to enroll and partake in the focus group data collection rather than being randomly sampled. Due to this self-selection, responses received may have been skewed due to strong pos-itive or negative thoughts on the study topic.

Likewise, the generalizability of the study data to other pregnant and postpartum women is limited as our sample size for this qualitative data collection was small. While our inclusion criteria were kept minimal to allow for data collection on a variety of motherhood journeys, gathering experiences from a more diverse population would allow for more robust representation of the subject. The women enrolled in this study were homogenous regarding income, education, race, and marital status. Data were not collected on mental health status or other behaviors (smoking, alcohol consump-tion, or medication) that may impact overall health outcomes. Collecting more com-prehensive data from a diverse population, e.g., in terms of socioeconomic status, would allow for understanding differences in access and privilege that play a role in health decision making.

## 5. Conclusions

Despite these limitations, the current study captured previously undocumented experiences of motherhood during the COVID-19 pandemic. Key findings highlight the importance of social support and social networks during pregnancy and postpartum. Many women also expressed concerns around their mental health and a lack of evidence-based resources on mental health and nutrition when trying to make healthy decisions. This novel feedback allows further insight into the needs of women, considering the unclear continuation of the pandemic, throughout pregnancy and postpartum.

The public health implications of this in-depth work during pregnancy and postpartum may help shape efforts to develop and implement new healthful eating and mental health communications during the first 1000 days. The current study provides basic quantitative data illustrating unhealthful eating and weight gain, which are putting pregnant women at greater risk of chronic disease; the qualitative data provide insight into the barriers women are facing when trying to live a healthy lifestyle during pregnancy and postpartum.

Healthy living throughout pregnancy and postpartum remains an important factor in the health and well-being of mother and offspring. This study highlighted that pregnant/postpartum women desire to lead a healthy lifestyle but experience barriers to accomplishing intended goals such as a lack of clear guidance on recommendations and a need for strong social networks. This formative work can prompt future studies to intervene through policy, systems, and environmental (PSE) interventions. Upstream resources and policies that promote healthy living for pregnant/postpartum women can reduce chronic disease throughout the lifespan following childbirth.

## Figures and Tables

**Table 1:** Focus Group Questions.

Pregnancy	Postpartum
Cognitive/Personal Factors (Knowledge, expectations, attitudes)	
What healthy eating advice do pregnant women need to know? What are your attitudes around eating behavior while pregnant? Can you describe places you have looked for eating advice while pregnant? How do you feel about healthy eating advice while pregnant? What areas of pregnancy caused you stress or worry?	What healthy eating advice do women who are recently postpartum need to know? What are your attitudes around eating behavior after having a baby? Can you describe places you have looked for eating advice since having a baby recently? How do you feel about healthy eating advice while postpartum?
Behavioral Factors (Skills, practice, self-efficacy)	
6. How much confidence do you have in choosing healthy foods? Can you describe why there is high/low confidence over healthy food choices? 7. Describe your skill level in preparing healthy meals. What barriers do you experience when trying to prepare healthy meals?	5. How much confidence do you have in choosing healthy foods? Can you describe why there is high/low confidence over healthy food choices? 6. Describe your skill level in preparing healthy meals. What barriers do you experience when trying to prepare healthy meals?
Environmental Factors (Social norms, access, influence on others)	
8. What factors (if any) in your environment make it challenging to eat healthy? 9. Describe any social support you get for healthy eating at home. 10. What would make your pregnancy a healthier experience? 11. In what ways has COVID-19 impacted your health or pregnancy?	7. What factors (if any) in your environment make it challenging to eat healthy? 8. Describe any social support you get for healthy eating at home. 9. What would make your postpartum journey a healthier experience? 10. In what ways has COVID-19 impacted your health or postpartum life?

**Table 2:** Descriptive Characteristics of Study Sample Pregnant and Postpartum Women.

Variable	Total	Pregnant	Postpartum	*p*-Value
Age (mean ± SD years)	57	29.18 ± 3.80	27.74 ± 3.16	0.1668
Marital status	57	18	39	0.3706
Single	3	0	3	
Married	53	18	35	
Widowed	1	0	1	
Race	57	18	39	1.0000 ^
White	56	18	38	
Other	1	0	1	
Residency	57	18	39	0.6312
Rural	29	10	19	
Urban or suburban	28	8	20	
Education Status	57	18	39	0.6435
High school or GED	7	3	4	
Associate degree (2 year) or some college	18	4	14	
Bachelor’s degree (4 year)	17	5	12	
Master’s or doctoral (PhD, JD, MD)	15	6	9	
Income	56	18	38	0.0587 ^
$10k to under 50k	6	0	6	
$50k to under $75k	12	3	9	
$75k to under $100k	15	4	11	
$100k to under $150k	17	10	7	
$150k to under $300k	6	1	5	
Gravidity (mean ± SD)	57	2.17 ± 1.12	2.05 ± 1.04	0.6072
BMI (kg/m^2^; mean ± SD) before pregnancy	55	26.45 ± 7.03	27.69 ± 6.87	0.3920
BMI (kg/m^2^; mean ± SD) since pregnancy	38	--	29.22 ± 6.98	--
sHEI (%; 0–100; mean ± SD)	57	53.98 ± 10.3	48.88 ± 9.94	0.0637

Wilcoxon rank sum test for continuous variables by pregnancy/postpartum group. ^ Fisher’s Exact Test for cell sizes less than 5. Body mass index (BMI); short healthy eating index (sHEI).

**Table 3:** Emergent themes for pregnant and postpartum women by SCT construct.

Theme, Subthemes	Supporting Quotes
Cognitive Factors	
1: Value and desire for healthy eating during pregnancy and postpartum	Something that we have changed is the products that we buy. We’re buying organic grass-fed milk and beef and free-range chickens and cage free eggs which before we would just kind of by like store brand, whatever was cheapest. I feel like they just taste better, but also, I feel like I’ve noticed, I just feel better when I eat it. So, I think it’s something that we’re going to stick with because we were talking about it and yeah, it’s more expensive, but at the same time you’re paying for the quality. It feels like it’s like its better quality, it tastes like its better quality. I just feel like if I’m doing it for her, why am I not doing it for me after? So, I think we’re going to stick with those habits that we’ve started to form (P). Besides trying to hit those guidelines, I [haven’t] really changed much in the way that I eat. It was more just kind of going through and trying to figure out specifically the breakdown of what’s in certain foods like I did not realize that I needed to eat more fiber because that was going to have benefits for [constipation]. It is a lot harder for you to digest and everything that goes along with that and changing the way that I eat would make other aspects of my pregnancy more comfortable (P). That was really important for me to know and then kind of just all the things that you’re not allowed to eat or that they say you’re not allowed to eat, because a lot of those when I actually looked into it, it was. Like you’re not supposed to eat it, but the guideline was from so long ago (P) In a good way from having the gestational diabetes, I learned so much about protein, and complex carbs versus simple carbs, all the really helpful information about all that stuff. So, I have carried that into now my boys’ diet, now that they both eat normal food like making sure they get really good protein, or I really need to make sure they get the complex carbs, trying to avoid the simple stuff, so that was a good thing that came out of like having that stupid gestational thing which was not fun, but it’s okay, it was a really good you know learning experience, so that was a good, positive effect from that (PP).
1.1: Influence on offspring’s health	I just to try to eat a variety of diets instead of just the same stuff so I have tried to open up and eat things that I wouldn’t usually eat healthy wise like I don’t usually eat many fruits, but I do try to do more of that stuff now to you know help with the health of the baby and I’m not really sure if it actually makes the difference in them and liking those foods, but I try (P). I’ve always been a very healthy eater in my adult life, at least not when I was a kid. So, for me super important just for me for my health and stuff that when I was pregnant to you know, I was eating as many fruits and vegetables as possible and, like even you know I would be like more careful (PP). I know that she doesn’t do very well with like excess caffeine, even though I feel like I needed in my life I’ve switched to decaf coffee and it seems to be working much better, especially for her. And I’ve also noticed that when I eat spicy foods, because I’m breastfeeding pretty exclusively like she’ll get a bottle every once awhile. But I noticed that spicy foods, she does not like those don’t bode well for her at all (PP). I’m a terrible cook, so yes, I’m trying to learn, I’m trying to grow, but I’m pretty terrible at it. So yes, that does affect how we eat. I still try—we just eat a lot of raw fruits and veggies, or I’ll just steam—I’ll get the frozen bags and steam them for veggies, and my sister says that’s just as good (PP).
1.2: Diet quality compromised in early postpartum	In the first few months it’s so important to, just like you said, survive. But it’s like reminding myself to eat when I’m feeding my boys. Meals were hard when they’re so young. Now that my boys, my youngest is only one, but he’s at the same eating schedule as my three-year-old, so we do breakfast, morning snack, lunch, afternoon snack, and then dinner. I eat the snacks too, so I eat five times a day. I do feel that the pandemic weight is sitting in because I just live in my kitchen. I try to count veggies and fruits and veggies for all of us. I try to get to five every day. But I fail at that. I maybe get like three days a week. Just the time, trying to eat with more convenience. When I was pregnant with my first, I had so much time to prepare for myself, and you know so just for the sake of like needing to get something quick and I don’t have time to just make something so fancy. But in terms of like attitude of like how I approach food since I’ve been pregnant versus postpartum. I was a little more strict and concerned and worried about what I would eat when I was pregnant (PP). I feel like those [three months] are so crucial and difficult that you really, literally just surviving is pretty much your goal, but once you get later, like I mean right now my son’s nine months, and I find that my … Because I’m also a stay-at-home mom, I find that my activity level is so much higher than it was when I was in an office. I’m having to eat a lot more to like to keep up because I know I need more calories per day, so I guess just like making sure that I’m making healthy choices, even though I’m you know grabbing something and going, you know from one thing to another all the time. Also, just breaking out of your normal routine because I’m a pretty routine-oriented person, so for me, it’s like breakfast, lunch, dinner, and now it’s like who knows when any meal is going to be (PP). When I first started breastfeeding, the hunger levels were like absolute like you’re an animal and I was eating as much as I could. [But] you always feel like you’re starving. When I was pregnant, I was trying so hard to follow my hunger cues. I was again so regimented and versus now is a lot, freer and a lot, you know a lot different. So I think just changing from eating, you know, whenever I was hungry while I was pregnant to go to going to breastfeeding when I was eating like probably 3000 calories a day and having even just to get used to that volume of food, you know, probably took a while (PP).
Behavioral Factors	
2: Desire to make well-informed health-based decisions	It’d be nicer if there was more availability and options for women for pregnancy safe or pregnancy friendly activity. I ended up paying for a subscription to a platform called move your bump that’s $20 a month and it’s entirely curated for women who are either trying to conceive, currently pregnant, or in their postpartum period. It’s run by [experts] So, all of them are very knowledgeable so that’s been amazing that I found it, but if I didn’t know about it, then I would have kind of been floundering this whole time (P). I went to the doctor, they were like well you don’t need to have like this increase of food, like the whole you’re not eating for two thing like is a myth and not real so they were like as long as you’re maintaining your normal diet, like you, eventually, like need to up like it was like a couple hundred calories but it’s not a lot, so that was nice for me to learn, because I was concerned in for a while that like I wasn’t eating enough (P). I don’t know if this is similar to other people, or if this is just because of the medical practice that I use, but I felt almost like at the beginning my pregnancy, like, I was floundering. I had to go find all this information on my own, because my doctor’s office didn’t give me anything. They kind of just gave me like a printed-out sheet that said, if you have this symptom take this medication, but not, these are the foods you should eat, these are the fruits you shouldn’t eat. So, I really had to go online or look through books. I didn’t appreciate the hands-off approach, especially with it being my first pregnancy there’s so many things that are overwhelming and so much information and not all of its accurate. Online anyone can put their opinion out there, so it’s annoying to have to sift through [information]. I am looking at someone’s blog where they’re telling me what they think or am I looking at a reputable website that is backed by knowledge and facts (P). My son couldn’t eat dairy, or soy. [We] went to a feeding team which had a dietitian. There were like six people in a room watching me feed my kid. And they kind of gave me a list of stuff not to eat, but then I never like me personally never had to follow up with them once we realized that feeding and was not the issue. I never went back to them (PP).
2.1: Need for comprehensive holistic approach to pregnancy and postpartum health	I feel like there’s been no emphasis on nutrition, there’s been no emphasis on mental health, they haven’t asked me about that at all at any of my appointments. So, I feel like that would be something that should be focused on for pregnant women, especially in the postpartum period. Because I’ve heard a lot of women say you know I had postpartum rage or anxiety, or depression and I was just told like “suck it up Buttercup it’ll get better, it’s just the baby blues”. I haven’t had any issues with it, yet, but I am aware that there’s kind of a lack of support if I do run into those issues (P). I feel like maybe if you know, I would have been more aware of like things that happen after you give birth. Like things happen so quickly and then like everything is constantly changing and like you have to be on like 24/7 now. So, it was hard to like to find this balance of like … Okay, what can I fit in this time and when am I ever going to sleep. And so, it was a really hard thing too. I personally struggled with trying to breastfeed for probably longer than I should have, to be honest. I felt like a failure but it’s okay like if it doesn’t work out, formula is fine. Your baby is fed, your baby is happy. It’s something I wish I [had] known just sooner. Yeah, I feel like that was the biggest part. I did have to then deal with some postpartum depression, and it was a lot (PP). I was back to work when COVID-19 hit, so I like I still get out and about to work every day. I had postpartum depression, and it was really bad. And I just like for three months I literally cried so I can’t imagine having COVID-19 at the same time, I don’t think I would have handled it well at all (PP). I think that has definitely like COVID has affected, my thoughts on going back to work, and especially like leaving her making sure that she’s safe and all of that so it’s affected a lot of stuff like my anxiety is definitely a lot higher because of things that I can’t control (PP). Seems like there’s a lot of focus on, eating to please baby or to make sure you’re avoiding allergies and not very much focus on like what you need to do to restore your own nutrient deficiencies, because I’m sure after (birth), you have nutrient deficiencies. Frankly. I don’t know very much about what I should be doing to fix that, if anything (PP). I would say, because I also didn’t have a lot of postpartum checkups, I had one at six weeks. They were kind of like all right you’re good to go. I’ve gotten really good care, but it’s kind of just like all right you’re good now we need to focus on the baby, which I get but also my body is a train wreck. I’m going back to work on Monday, 10 weeks postpartum and generally, I feel good but I still feel like there are so many things that are happening in my body that I don’t know what’s going (PP).
2.2: Ability to navigate the internet and social media to access desired information	There’s a website that my doctor’s office gave me [where] you could type in any food or medication, and it would tell you what the current recommendations are from American Pregnancy Association. But I went to this website and I just it wasn’t very user friendly. You have to type in what your question is and then someone emails you back, but the email responses were so snarky that I never wanted to use it again. So now what I’ve been using the most is the Mayo clinic guide to a healthy pregnancy, so that had some good information in it, and then I use the *Ovia* pregnancy application, and I’ve used the ‘what to expect’ website a bunch. Both of those have been very helpful (P). I feel like I’m the type of person that Google’s everything I mean, I know how to sift through information too, but sometimes it’s hard because there’s a lot of like contradicting stuff (PP). I remember scrolling through [Instagram and Pinterest] and trying to save charts that would have like these things are good to help breastfeeding like oats and almonds and that kind of stuff and all you know the fruits and veggies. So yes, okay, so I did do that to boost my (supply) (PP). I also got a lot of information from Facebook groups of other moms that had kids the same month as me, and on Reddit, they also have like groups, private groups for people who are due in a specific month, so it was kind of nice to bounce ideas off of other women who were in the same stage of postpartum (PP). I went in search of it, I knew they existed. I joined it on bump.com and then it switched to a private Facebook group (PP). Well, for me, I’m in like a couple like Facebook groups for healthy recipes and things like that—I don’t really get caught up in like the fad diets, I am just trying to replace [foods] with healthy things that I find or whatever seems healthy, I guess. For my son, I’m following a yummy toddler food or the solid starts Instagram [page], so I’m trying to base things off of that (PP).
Environmental Factors	
3: The role of social networks during pregnancy and postpartum	Talk to your doctor [about] anything. It is valuable because your doctor is better able to understand what your diet is currently like and if there is anything major that needs to change. I think there that can be a lot of like shame that goes into how you eat during pregnancy, to be like a terrible mom because I’m not eating healthy enough (P). My husband is great, and he cooks many almost all of our dinners and he is a very healthy eater and so that has helped me to eat healthy. It’s easy when I don’t have to do it (P). My best friend is pregnant right now to she’s eight weeks ahead of me, so it’s been amazing going through this. She’s on the east coast, unfortunately for me. So, we have not seen each other, but we are able to talk and kind of, say, like oh hey this is going on, did you go through this, how did you deal with that when you went through it as well, so that has been nice (P). I work at a pharmacy so everyone there is fairly healthy and all about clean eating as well, and being healthy, so that everyone, there is a big influence as well to stay on the right track, and we all try to get together and you know talk about healthy eating and just you know we have a water machine there so that’s a big thing of ours, we all go to the water machine drink plenty of water all day (P). I just recommend people to ask for help. Ask for help, whether it be you know nutrition wise trying to help get meals together recipes that are healthy, yet still quick and convenient to make whenever you know you’re fresh out of the hospital and all that stuff is new that would be something helpful (PP). I definitely feel influenced at work, everyone [will] be like ‘hey let’s order pizza for lunch’ or ‘let’s all get this.’ There are people in office they eat healthier but it definitely [influences] the things I eat. At home now my husband will eat whatever, but my three-year-old eats absolutely nothing so I’m constantly trying to figure out different ways to make something that he’ll eat (PP). I’m trying to think like my sister now because she’s so knowledgeable and honestly my parents because they cooked pretty well for us growing up, but like I don’t go, I don’t, I don’t really go searching out for nutrition advice, or I’ll just Google it. If I have a question, I’ll just Google it, or I’ll ask my sister (PP). I found out of this because of a virtual group that I am in. When I first found out I was pregnant, I found a Reddit group initially that were all 2020 bumpers. So, like that, like it did like form into like a Facebook group, and so you know we it’s like our big Community village, I would not have survived anything without this group, it has been like such a blessing, and I am so thankful (PP).
3.1: COVID-19 pandemic impact on social networks	COVID-19 has impacted my pregnancy, doctor appointments in general, no one’s really allowed to go. Just like special events and occasions with family and friends trying to celebrate the baby, that has really put all that on a tight hold and just trying to social distance and you know kind of go at it, with just your family here at home. It can be tough. (P) The first three months, especially because it was my first and we did not have … We were in quarantine, so we didn’t have parents coming over and staying with us, we didn’t have a babysitter, we did not have, you know, anything like that. So, we were like 100% on our own for those first, I mean kind of still a little bit, but for those first three months, because he was born in April, so it was that was probably the most stressful time for us, I would say. I think a lot of we had a lot of food drop-offs, and those really were like a saving grace with just people being able to do that. He’s actually not seen anyone, except for me and his dad without a mask on (PP). It was kind of intense and still is like I said we didn’t have any help we actually, even the hospital situation we couldn’t leave if we left the hospital nobody could come back luckily my husband was allowed to be there with me, but we could not have any visitors there …, I wanted to do like music classes and like bring on playdates with other kids and meet for coffee, and you know even go to the grocery store and have people be like oh your baby is so cute. Like I’ve never had any of that, and so it is an adjustment for my mental health (PP).

P = pregnant women; PP = postpartum women; COVID-19 = coronavirus 2019.

## Data Availability

Data is available from the PI upon request.

## Supplementary Materials

The following supporting information can be downloaded at: https://www.mdpi.com/article/10.3390/ijerph19105849/s1, Figure S1: Study Design Flow Chart.
