# Supplementary figures and images for: “There’s a Lot of Like, Contradicting Stuff”—Views on Healthy Living during Pregnancy and Postpartum
Source: Int J Environ Res Public Health. 2022 May 11;19(10):5849. doi: 10.3390/ijerph19105849 (PMC9140655; doi:10.3390/ijerph19105849)

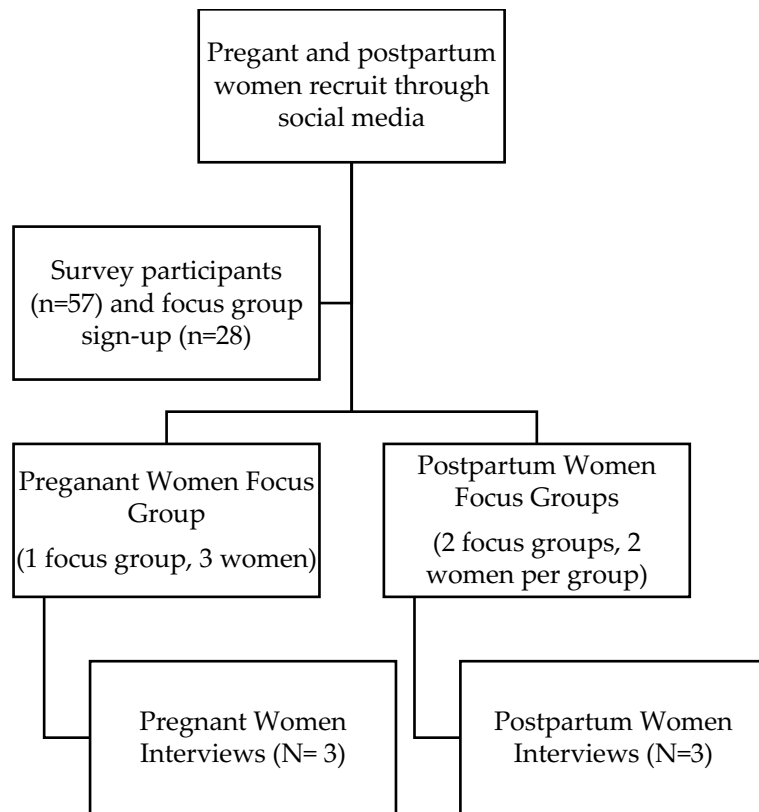

Figure S1: Study Design Flow Chart

Supplement: Supplementary file 1 [file ijerph-19-05849-s001.zip › ijerph-1673941-supplementary.pdf]
